# Overcoming Extraction Hurdles and Assessing Biological Activity in a Major Invasive Seaweed Species in Europe, *Rugulopteryx okamurae*

**DOI:** 10.3390/md23040141

**Published:** 2025-03-25

**Authors:** Carolina Paulo, Joana Matos, Cláudia Afonso, Carlos Cardoso

**Affiliations:** 1 Division of Aquaculture and Upgrading (DivAV), Portuguese Institute for the Sea and Atmosphere (IPMA, IP), Avenida Alfredo Magalhães Ramalho, 6, 1495-006 Lisbon, Portugal; 202100204@estudantes.ips.pt (C.P.); joana.matos@ipma.pt (J.M.); cafonso@ipma.pt (C.A.); 2 Polytechnic Institute of Setúbal, Rua Américo da Silva Marinho, s/n, 2839-001 Lavradio, Portugal; 3 CIIMAR, Interdisciplinary Centre of Marine and Environmental Research, University of Porto, Rua dos Bragas 289, 4050-123 Porto, Portugal

**Keywords:** *Rugulopteryx okamurae*, invasive seaweed species, biologically active compounds, antioxidant activity, anti-inflammatory activity, process optimization

## Abstract

The brown seaweed *Rugulopteryx okamurae* is a major invasive species in Europe, menacing local ecosystems. The challenge lies in assessing application routes for this biomass, testing different extraction technologies (overnight agitation, mechanical homogenization, pH-shift, ionic liquid-, and ultrasound-assisted extractions) and parameters. There was a higher yield in the extracts homogenized with 70% ethanol, especially with 1:20, *w*/*v*, biomass–solvent ratio, than in aqueous extracts. As to overnight agitation, 70% ethanol produced results (24.5–28.3%) similar to those found in the homogenized extracts. However, in the former, the best biomass–solvent proportion was 1:10, *w*/*v*. Mineral matter yield presented specific patterns, reaching 59.6 ± 1.1% (70% ethanol) and 82.3 ± 0.1% (water). The highest total polyphenol level was attained in the 70% ethanol, 1:20, *w*/*v*, extract, 310.7 ± 22.1 mg GAE/100 g dw seaweed. This extract had a higher FRAP/ABTS. The extract attained with overnight agitation, 70% ethanol, 1:10, *w*/*v*, had 48% COX-2 inhibition as anti-inflammatory activity. Besides showing the potential of *R. okamurae* for pharmacological purposes, especially in the antioxidant and anti-inflammatory area, this study enabled us to rank technologies and conditions for the utilization of this abundant biomass resource by the industry.

## 1. Introduction

The brown seaweed *Rugulopteryx okamurae* is a major invasive species in Europe. This alga belongs to the Class Phaeophyceae, Order Dictyotales, and the Family Dictyotaceae, originating from the Pacific Ocean, especially the coasts of China, Korea, and Japan [1]. This species was first spotted in France, afterwards spreading along the French and Spanish Mediterranean Coasts and has reached the Atlantic Ocean, including Portuguese shores, namely Algarve and São Miguel Island in the Azores Archipelago [2]. Moreover, its exponential expansion in the Western Mediterranean and Portuguese Southern Coast has been accompanied by intense blooms, that is, phases of very intense growth with enormous biomass generation. After declining and dying, this generates huge deposition on the shores and vast environmental impacts on the beaches of southern Portugal, particularly in the Algarve, a major tourist destination in the country. In fact, this invasive species is menacing local ecosystems with the possibility of causing a future irreversible situation [3] with major effects on human activities. Therefore, measures aiming at minimizing such effects are urgent and may encompass various socio-economic sectors. Indeed, although this invasive phenomenon is quite recent, research on potential applications for this algal biomass has already started as a foundation for a strategy to minimize the accumulation of *R. okamurae* waste on the coast and the costs associated with its removal and elimination [4]. However, this research is still incipient [3].

The biomass of *R. okamurae* may harbor valuable components, including biologically active substances. It has been noted that the invasive success of this species on the shores of Southwest Europe may be due to the potential presence of chemical defenses [3]. A recent study [5] was able to identify relevant secondary metabolites of *R. okamurae* collected on the coast of the Strait of Gibraltar, namely detecting diterpenoids, such as dilkamural. This compound was deemed to be a major chemical component with anti-herbivore action and decisively involved in the invasive ability of *R. okamurae* [5]. The algal biomass contained the remarkable concentration of 4.21 ± 0.39%, w/dw, dilkamural [5]. This highlights the particular biochemistry of this seaweed species, at least in what concerns its adaptation to the European environment. It also raises the question of the existence of other specific components in its biomass, as is the case in other brown seaweeds known to be rich in peptides as well as sterols, polyphenols, flavonoids, and many other secondary metabolites and to display various biological activities, including antioxidant, anti-inflammatory, antifungal, antibacterial, and anti-tumour properties [6]. Due to insufficient research, there are still few studies on the possible applications for the biomass of *R. okamurae* [3,7]. Such studies refer mostly to its use in anaerobic bio-fertilizer production, the creation of bio-plastic materials, but also its application in the field of biostimulants and supplements in aquafeed. According to these and other authors [7,8,9,10], *R. okamurae* usually shows low levels of phenolic compounds, which may limit its potential in comparison to other brown seaweed species. However, there are some results pointing to the possible antimicrobial and anti-inflammatory activities of its biomass, which may pave the way for biomedical or nutraceutical applications for specific biomass fractions/extracts [11].

In order to attain such extracts, it is necessary to overcome extractive hurdles that are often intrinsic to seaweed biomass [12]. Indeed, extracting the biologically active compounds and achieving extracts with high activity is a key challenge in many types of seaweed [12,13]. Moreover, these extractive processes should be integrated in a whole strategy of biomass valorization, the so-called biorefinery approach. In the case of *R. okamurae*, there are very few studies on this subject [14,15]. Namely, Fernández-Medina et al. (2022) [14] studied the application of enzyme hydrolysis and dark fermentation to the biomass of *R. okamurae*. They also analysed the effect of a microwave pretreatment and found out that it is positive for the solubilization of organic matter in *R. okamurae*, specifically in the 180–220 °C range. On the other hand, López-Hortas et al. (2023) [15] optimized a microwave hydrodiffusion and gravity treatment and found it advantageous in recovering liquid fractions with high yields and antioxidant potential when compared to those obtained after ethanolic solid–liquid extraction of conventionally dried seaweed.

Nonetheless, this subject is far from thoroughly studied and fully understood. It may be conjectured that the cell walls of *R. okamurae* are difficult to disrupt in order to extract cell contents, it being known that carbohydrates make up roughly 60% of its composition on a dry weight basis, with alginate being the main constituent, reaching approximately 32% of the total composition [16]. In other brown seaweeds, the cell wall usually contains cellulose in small amounts, being composed of at least two different layers. In fact, whereas the inner layer is chiefly constituted by cellulose, which imparts rigidity to the wall, the outer layer is an amorphous matrix made up of alginate and fucoidan, thus ensuring a combination of strength and flexibility in the cell wall [17,18]. Given the difficulties associated with such structural features and polysaccharides, various extractive technologies have been applied to seaweed biomass by different authors [19,20]. Some methodologies may be advantageous in that they are more operative in disrupting cell walls and releasing cell contents, but may also be negative if they degrade more sensitive—namely more thermolabile or oxidizable—compounds [20,21,22]. Hence, alternative methodologies have been suggested for an improved process performance, but also for a smaller environmental impact and higher safety for consumers. In particular, the utilization of green solvents, ranging from water to isoamyl acetate, which are solvents that are neither toxic nor generate toxic substances that are harmful to the environment, has been proposed [23].

Therefore, the performed experimental work aimed to assess the potential of extracting contents and specific biologically active compounds from the biomass of the invasive seaweed *R. okamurae* and, also, to compare different technological processes (including different green solvents) and the respective operational parameters in achieving extracts with optimal properties.

## 2. Results

### 2.1. Dry Matter and Mineral Matter Yields

The total dry and mineral matter yields (Table 1) were calculated (see Section 4.4. “Determination of Moisture and Ash and Calculation of Yields”) using the determination of moisture and ash contents in the initial biomass of *R. okamurae* and in the various extracts.

With respect to the dry matter yield, values differed widely, especially as a function of the type of extractive methodology. Extremely low yields, not exceeding 6%, were observed in all cases with ethyl and isoamyl acetate using mechanical homogenization (H) as well as with the ionic liquid (IL). The utilization of water or a 70%/30% ethanol–water mixture in H brought a significant improvement to the yield, thereby reaching 24.4–29.8%. There was a better extractive performance in the Hew trials, especially with 1:20, *w*/*v*, biomass–solvent proportion, in comparison to the Hw ones. Moreover, while the pH-shift technique brought forth low yields in the 13.3–14.5% range, the UA trials led to intermediate values in the 19.6–21.6% gamut. In the case of the OA trials using a 70%/30% ethanol–water mixture, there were results (24.5–28.3%) similar to those found in the H extracts. However, in the former, the best biomass–solvent proportion was 1:10, *w*/*v*, different from the H trials.

On the other hand, despite some similarities with the dry matter yield, the mineral matter yield showed a different range of levels and patterns. Concerning similarities, the H trials using ethyl and isoamyl acetate and the IL trials had poor results, with no detectable extraction of mineral matter. Furthermore, on the other extreme of the yield range, the H trials using water and a 70%/30% ethanol–water mixture achieved yields between 59.6 ± 1.1% (Hew1:20) and 82.3 ± 0.1% (Hw1:20). There were significant differences between water and the 70% ethanol extracts, delivering the former higher mineral matter yields. The same phenomenon was seen in the UA trials, with the water extracts presenting 53.4–60.3% yields that were higher than those of the UAew extracts, 26.9–33.9%. In addition, for the same biomass–solvent proportion, the OA trials displayed a similar pattern. Namely, whereas the OAw1:10 had a 64.7 ± 0.4% mineral matter yield, the OAew1:10 was lower with 54.0 ± 4.2%. Finally, it is worth mentioning that the pH-shift extractions presented relatively high yields (64.4–64.8%), thereby equalling the best of the OA and UA trials, but falling behind the H ones with water.

### 2.2. Total Polyphenol Content

Besides yields, regarding all studied methodologies (including all tested operational parameters), the total polyphenol levels in the attained extracts were also analysed, and are presented in Table 2.

Just as for the dry matter and mineral matter yields, there is a division of extractive methodologies according to their efficiency. The IL (ionic liquid) and pHS (pH-shift) values as well as those from the Hea1:10 trial (using mechanical homogenization) were low, not exceeding 100 mg GAE/100 g dw. There were intermediate values between 100 and 200 mg GAE/100 g dw for many other extracts and five trials that resulted in total polyphenol levels above 200 mg GAE/100 g dw. The highest result was achieved in the Hew1:20 trial, 310.7 ± 22.1 mg GAE/100 g dw and homogenizing *R. okamurae* biomass with 70% ethanol at a lower ratio (1:10, *w*/*v*) also led to a high polyphenol concentration, 255.6 ± 12.8 mg GAE/100 g dw. This was similar to results also attained with 70% ethanol, but by overnight agitation (240.0–260.7 mg GAE/100 g dw) or ultrasound-assisted extraction (192.1–236.5 mg GAE/100 g dw). Moreover, the utilization of water as an extracting medium in the mechanical homogenization trials did not cause a meaningful difference to ethyl or isoamyl acetate. It can also be noted that while increasing the biomass–solvent ratio did not alter significantly the polyphenol content in the Hw trials, it improved this parameter in the cases of ethyl and isoamyl acetate and, also, of ethanol 70%. With respect to the OA batches, a distinctive positive effect of the biomass–solvent ratio as well as of using ethanol 70% instead of water on the polyphenol content was clearly observed. Finally, in the UA trials, whereas the biomass–solvent ratio did not make a difference, for each fixed ratio, there were large increases (up to 80%) in phenolic levels with the replacement of water by ethanol 70%.

### 2.3. Biological Activity

The extracts that showed the best dry/mineral matter yields and highest polyphenol contents (OAew1:10 and Hew1:20) were chosen for further study. Accordingly, the biological activity in these extracts was subjected to a more detailed analysis, thereby comprising determination of the antioxidant activity by three distinct methods, DPPH, FRAP, and ABTS techniques, leading to the results shown in Table 3, Table 4, and Table 5, respectively, and correlating these activities with the polyphenol content (Pearson coefficients displayed in Table 6).

Regarding DPPH, mechanical homogenization (Hew1:20) led to higher antioxidant activity than overnight agitation (OAew1:10), 40.8 ± 0.5 mg AA Eq./100 g dw vs. 18.8 ± 0.1 mg AA Eq./100 g dw. This same pattern was also observed in the case of the two other methods for measurement of the antioxidant properties, FRAP and ABTS. Though the increment of antioxidant activity from OAew1:10 to Hew1:20 in these cases was not so large, it was still quite significant, in the 40–60% range. Concerning the anti-inflammatory activity, the OAew1:10 displayed a 48.3 ± 7.1% level of COX-2 inhibition.

## 3. Discussion

### 3.1. Dry Matter and Mineral Matter Yields

Regarding extractive yields, the comparison between methods and operation conditions and parameters is of paramount importance in optimizing seaweed extraction and achieving its valorization [24,25,26]. In fact, the yields (total extracted matter from seaweed) reported in the literature vary widely as a function of the type of seaweed, extraction technique, and operational parameters [24,25,26]. Concerning dry matter yield, seaweed type seems to be a key factor, since Rodrigues et al. (2015) [26] observed lower yields with a brown seaweed species, *Sargassum muticum*, never exceeding 32% in their comparison between hot water, ultrasound-assisted, and enzyme-assisted extractions. This seems to be also the case in the current study of *R. okamurae*, suggesting difficulties in totally disrupting cell walls and releasing contents.

The best yield results were achieved with mechanical homogenization and overnight agitation. However, in the former case, there were large differences as a function of the utilized solvent. Indeed, the homogenization of seaweed biomass may present difficulties leading to lower yields. On the one hand, it may help in disrupting the cell wall and increasing the surface area for a more efficient mass transfer of solutes present in the algal cells. On the other hand, a negative impact on yield may result from adsorption phenomena to a larger surface area of the smaller particles after homogenization—particularly when the extracting solvents have low chemical affinity with the targeted compounds, as seems to happen with ethyl and isoamyl acetate. In the case of *R. okamurae*, if more polar solvents, such as water and 70% ethanol, are used, dry matter and mineral yields are markedly improved, an indication that the positive effect of homogenization on mass transfer outweighs the aforementioned adsorption phenomena. Though, to the best knowledge of the authors, this specific form of homogenization was not previously applied to *R. okamurae* biomass in an extractive perspective; it can be compared with recent milling experiments performed by De La Lama-Calvente et al. (2024) [27] using *R. okamurae*. These authors concluded that a cascade system where the biomass is firstly extracted with ethanol and then with water, or vice versa, would be the best approach to augment extraction yields. The results of utilizing ethanol 70% in the homogenizing process (and in the OA technique) in the current study seem to converge with this observation.

The UA technique may also be advantageous, since it is a cold extraction technique, provided that exposure time to ultrasounds is not too long. This may ensure the stability of the targeted molecules, while decreasing or excluding the use of toxic chemicals and enhancing the yield and quality of the intended products [28]. Ultrasound-assisted extraction takes advantage of acoustic cavitation and associated physical phenomena, which depend on UA frequency, time, and other factors [29]. Besides fragmentation/erosion, which may lead to difficulties as mentioned regarding the H technique, ultrasounds can enhance sonocapillary—a stronger penetration of solvent into the canals/pores of the matrix [29]—and sonoporation—a higher permeability of cell membranes—thereby improving the release of intracellular contents by opening membrane pores [30]. Though UA conditions used with *R. okamurae* were relatively mild, results seem to be poorer than those attained with homogenization. Hence, the used conditions may have failed to prevent excessive fragmentation/erosion or sonocapillary and sonoporation phenomena were not strong enough to enable higher yields. Other researchers working on ultrasound-assisted extraction and seaweed biomass, such as Le Guillard et al. (2016) [31], achieved higher yields with an ultrasound frequency of 35 kHz, similar to that applied to *R. okamurae*, 40 kHz, but a much longer time, 6 h vs. 40 min. Specifically concerning UA and *R. okamurae*, a very recent study by León-Marcos et al. (2024) [32] showed that 100% amplitude and 4% (*w*/*v*) algal concentration—similar to 1:20, *w*/*v*, in the current study—led to optimal results, ensuring the maximum Chemical Oxygen Demand (COD) solubilization of 61.5 mg COD/g dw *R. okamurae*. This is still a relatively modest value, but these authors used ultrasound technology as a previous treatment to biological processing.

Finally, extraction methodologies, which are usually less applied to seaweed, such as pH-shift, seem to be inefficient in extracting total organic matter, being more selective and adequate to specific solutes, such as protein [33]. Likewise, extraction with an ionic liquid did not work in *R. okamurae*, its ability to efficiently dissolve cellulose [34] being of little use in this case.

### 3.2. Total Polyphenol Content

There is some degree of agreement between the total polyphenol contents and the dry matter yield results (Table 1). For instance, low dry matter yields in the pHS trials matching equally low phenolic contents and higher yields in the Hew trials are coupled to high polyphenol levels. Accordingly, total polyphenol levels support the pattern observed in the case of the dry matter yields and highlight a certain degree of similarity in the mechanisms underlying polyphenol and overall organic matter extraction. From an industrial point of view, this is also relevant because it informs any production engineer that strives to optimize phenolic extraction about a possible proxy for quickly converging toward the most suitable extraction conditions.

Polyphenol results diverge from previous studies on different brown seaweed species comparing hydrophilic and hydrophobic extractions [35,36], and did not point to higher phenolic contents in the extracts of more apolar solvents, such as 70% ethanol. However, there are other studies [27,37], including on *R. okamurae*, that favour more apolar solvents and indicate higher phenolic yields with ethanol in comparison to water. For instance, De La Lama-Calvente et al. (2024) [27] could achieve phenolic yields with ethanol that were threefold those in water extracts using mild temperatures and short milling times. In any case, the largest differences in polyphenol yields were due to the application of alternative extractive technologies. In the cases of OA, H, and UA (with the exception of Hea1:10) technologies, measured phenolic contents agree with the published literature for an algal biomass rich in these substances, usually displaying values between 100 and 500 mg GAE/100 g dw [37,38]. In particular, Belhadj et al. (2024) [37] determined a total phenolic content of 270 ± 20 mg GAE/100 g dw in a 70% ethanol extract (microwave-assisted extraction) from *R. okamurae* collected in May on European shores (Gibraltar). This is similar to the best results using 70% ethanol in the current study, at least in the cases of OA and H techniques.

### 3.3. Biological Activity

In the case of antioxidant activity, different methodologies focus on different antioxidant pathways and, as such, may widely diverge in their results. However, this was not the case in the current study. Indeed, while ABTS is deemed more sensitive than DPPH and responsive to compounds with different chemical affinities [38,39], all three methodologies pointed to more powerful antioxidant properties in the *R. okamurae* extract attained by mechanical homogenization (Hew1:20) than in the other attained by overnight agitation. Moreover, a comparison to the study carried out by Belhadj et al. (2024) [37] in European *R. okamurae* shows a similarity to the Algarve samples in what concerns the absolute antioxidant activity levels. Namely, Belhadj et al. (2024) [37] reported an ABTS level of approximately 1800 μmol Trolox Eq./100 g dw in a 70% ethanol extract (microwave-assisted extraction), which compares to 1489 μmol Trolox Eq./100 g dw in the Algarve *R. okamurae* (Hew1:20).

It must be remarked that Hew1:20 also presented the highest total polyphenol content. It is well-known that polyphenols are effective antioxidants [40], being divided into phenolic acids, flavonoids, stilbenes, lignans, tannins, phlorotannins, and other groups. The matrix of Pearson coefficients (Table 6) highlights a high degree of correlation between total polyphenol content and antioxidant activity levels, regardless of the particular method used. Only antioxidant activity measured by DPPH suggests a non-negligible contribution of compounds other than polyphenols. Within phenolic substances, phlorotannins are the major group in brown seaweeds, being constituted by chains of 1,3,5-trihydroxybenzene (synthesized by the acetate–malonate pathway) and displaying a wide range of molecular weights, 126–65,000 Da [41]. Phlorotannins isolated from brown seaweeds have been reported to exhibit relevant bioactive properties, such as antioxidant, anti-cancer, anti-inflammatory, or anti-allergic activities [42]. López-Hortas et al. (2023) [15] were able to quantify the total phlorotannin content in an ethanolic extract of *R. okamurae*, 200–300 mg Phloroglucinol Equivalent/100 g dw. Moreover, there are pigments (e.g., carotenoids) and other molecules in the algal biomass with antioxidant properties [43] that may also have contributed to the high antioxidant activity in the selected extracts, particularly in Hew1:20.

With respect to the anti-inflammatory activity, besides study scarcity, a literature comparison is rendered more difficult due to the diversity of determination methods, which comprise in vitro and in vivo assays [44,45,46]. Cuevas et al. (2021; 2023) [11,47] used the inhibition of nitric oxide production as a method to assess anti-inflammatory activity in *R. okamurae* and were able to quantify a substantial activity level as well as to prove the existence of specific anti-inflammatory compounds in the algal biomass. These belonged to the diterpenoid class [11,47]. Regarding inhibition of the COX-2 activity by *R. okamurae* extracts, to the best knowledge of the authors, no previous study has been carried out for this new species on the European shores. Accordingly, a direct comparison—applying exactly the same anti-inflammatory assay—is only possible with other brown seaweed species, such as *Halopteris scoparia* and *Petalonia binghamiae*, whose ethanolic and aqueous extracts, respectively, have substantial anti-inflammatory activity [35]. Indeed, a COX-2 inhibition of 79 ± 8% was determined in the ethanolic extract of *H. scoparia*. Though the activity level in the 70% ethanol extract of *R. okamurae* subjected to overnight agitation (OAew1:10) was more modest, it was still significant and could be higher with a 96% ethanol extraction as carried out by Campos et al. (2019) [35]. Besides diterpenoids, it is possible that the phlorotannins (polyphenols) in OAew1:10 were influential for the anti-inflammatory properties [42], it being known that there is a large variety of compounds in *R. okamurae* biomass with a potential for multiple biological activities [9].

These biological activity results highlight the potential of *R. okamurae* for pharmacological purposes, especially in the anti-inflammatory area, due to the presence of a varied array of compounds. Considering that *R. okamurae* is an invasive species in Europe with huge levels of biomass production that have highly deleterious effects on the region [48], this could be an opportunity to economically support the wild harvest of this species to use it in valuable applications [11].

## 4. Materials and Methods

### 4.1. Experimental Design

**Figure 1:**
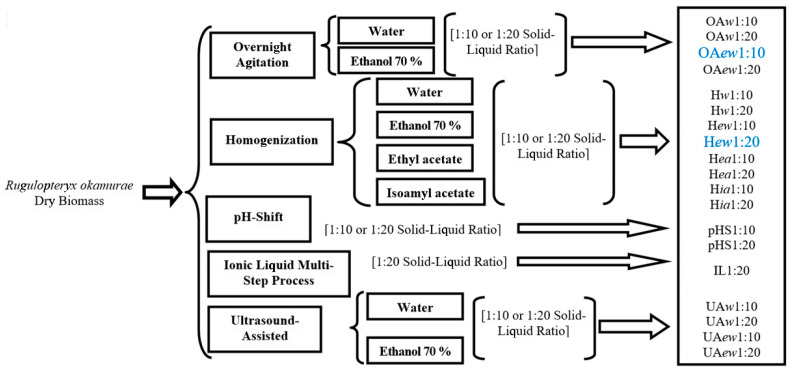
Experimental design of the study on the comparison of extraction methodologies (selected ones are indicated by blue codes in larger font).

The experimental work was designed with the purpose to compare different methods and technological parameters for the optimal extraction of target compounds with antioxidant and/or anti-inflammatory activities from dry *R. okamurae* biomass. The studied methods and parameters were the following: (i) conventional solid–liquid extraction with overnight agitation (coded as OA and using water, w, or ethanol–water 7:3, ew); (ii) solid–liquid extraction with intense mechanical homogenization (drastic physical process, coded as H) and encompassing so-called green solvents (water, w, ethanol–water 7:3, ew, ethyl acetate, ea, and isoamyl acetate, ia); (iii) pH-shift extraction (coded pHS); (iv) ionic liquid multi-step extraction (IL); and (v) ultrasound-assisted extraction (UAw, using water, or UAew, using ethanol–water 7:3). Different proportions of seaweed biomass to extracting liquid (1:10 or 1:20, *w*/*v*) were tested. All these methods and operational parameters (Figure 1) were used with the purpose to optimize the extractive process, taking the most usual route process as the control (solid–liquid extraction with overnight agitation).

Following extract preparation, moisture and ash contents were determined and used to calculate the yields in terms of dry and mineral matter extraction. Total polyphenol content was also determined for all extracts and expressed per weight of dry seaweed. Afterwards, the best extracts, that is, those with higher yields and total polyphenol content, were selected for a deeper characterization of their biological activity. The overall experimental scheme and detailed codes are shown in Figure 1.

### 4.2. Seaweed Source, Collection, and Preparation

The studied seaweed was a brown species (Class Phaeophyceae), *Rugulopteryx okamurae*, which is not native to Portuguese waters or even European shores and is considered invasive. The samples used in this study were collected by the company Easy Harvest (Faro, Algarve, Portugal) on a beach in the Barlavento Algarvio (37.11 °N; 8.53 °W) following an episode of massive deposition of algal biomass on that beach in March 2023. The taxonomic identification was ensured by the supplier. Briefly, its phenotypical features were observed; namely, an external vegetative morphology of thalli that was flat ribbon-like corrugated and dichotomously branched up to 18 cm height with a stoloniferous attaching system, exhibiting a yellow-brown colour without iridescence, was verified. Moreover, the inner structure of the thalli was characterized by a monostromatic cortical, a monostromatic central medulla, and a pluristromatic medulla at the margins, thus further confirming identification, as previously completed at other locations [49].

After harvesting the *R. okamurae* seaweed (approximate total of 20 kg), it was transported to an Easy Harvest company facility and immediately subjected to a drying process in a solar oven. This process lasted three days and resulted in mass loss through evaporation (final mass of approximately 3 kg). Drying was followed by grinding the algae in a chopper, which ended with the reduction of dry biomass to particles <1 mm in size. This dry powder was packaged and later sent to the IPMA laboratory in Lisbon. Immediately after reception, seaweed biomass was put inside vacuum-packaged bags at −80 °C in a Thermo Scientific 88000 Series freezer (Thermo Fisher Scientific, Inc., Waltham, MA, USA) and stored at this temperature until further processing.

### 4.3. Seaweed Extraction Methodologies

A set of five different extractive methods were applied to *R. okamurae*, based on physical processes and/or chemical treatments. In the case of the former, overnight agitation, mechanical homogenization with a high velocity rotating disperser rod, and ultrasound-assisted extraction were applied. As to chemical processes, pH-shift and ionic liquid multi-step extraction were tested.

#### 4.3.1. Overnight Agitation (OA)

This is the most usual solid–liquid extraction process and involves the addition of a hydrophilic or hydrophobic solvent to dried seaweed at a given proportion and a following lengthy agitation step [50]. In this study, water (w) and ethanol–water (70%, *v*/*v*) (ew) were added either at 1:10 or 1:20 ratios, *w*/*v*, to dry *R. okamurae* (OAw1:10, OAw1:20, OAew1:10, and OAew1:20). For this extraction, 5 g of *R. okamurae* was weighed in an OHAUS EX224M analytical balance (Parsippany-Troy Hills, NJ, USA) and then the required volume of solvent was added. After preparing all extracts, the beakers containing the solid–liquid mixtures were placed in an IKA KS260 orbital shaker (IKA-Werke GmbH & Co. KG, Staufen, Germany) and left shaking (200 rpm) overnight at 20 °C. The following day, extracts were transferred to tubes and centrifuged in a Kubota 6800 centrifuge (Kubota, Osaka, Japan) at 3000× *g* and 20 °C for 10 min. Subsequently, supernatant (extract) was transferred to new tubes and stored in the refrigerator at 4 °C until analysis.

#### 4.3.2. Homogenization (H)

Dry *R. okamurae* biomass was used for this extraction procedure and this solid–liquid technique [51] used four alternative green solvents (water, ethanol–water, 70%, *v*/*v*, ethyl acetate, and isoamyl acetate) added either at a 1:10 or 1:20 ratio, *w*/*v*, to the biomass (Hw1:10, Hw1:20, Hew1:10, Hew1:20, Hea1:10, Hea1:20, Hia1:10, and Hia1:20). Briefly, 5 g of dry *R. okamurae* was weighed in an analytical balance and the required volume of solvent was added. Then, the prepared mixtures were homogenized using a CAT Unidrive X1000D homogenizer equipped with a large diameter rod (Ingenieurbüro CAT, M. Zipperer GmbH, Ballrechten-Dottingen, Germany). After three 5-min cycles at 30,000 rpm, the solutions were transferred to centrifuge tubes and centrifuged at 3000× *g* and 20 °C for 10 min. Afterwards, the supernatant was transferred to new tubes and stored in the refrigerator at 4 °C until analysis.

#### 4.3.3. pH-Shift (pHS)

Dry *R. okamurae* biomass was also used in the pH-shift extractive method. This method entails a solid–liquid extraction with an acidic aqueous solution followed by a pH neutralization step [52]. The acidic solution is added either at a 1:10 or 1:20 ratio, *w*/*v*, to the biomass (pHS1:10 and pHS1:20). For this purpose, 5 g of dry *R. okamurae* was weighed in an analytical balance and the amount of 1 M HCl necessary to obtain the desired proportions was added. Then, the attained solid–liquid mixtures were placed in an orbital shaker and left shaking (200 rpm) overnight. The following day, pH level of the mixture was measured using a Mettler-Toledo SevenCompact S220 pH meter (Mettler-Toledo GmbH, Greifensee, Switzerland) and the extracting mixture was carefully neutralized by adding 5 M NaOH solution dropwise until a pH between 6.8 and 7.2 was obtained. After neutralization, the tubes were centrifuged at 3000× *g* and 20 °C for 10 min. Finally, the supernatant was transferred to new tubes and stored in the refrigerator at 4 °C until analysis.

#### 4.3.4. Ionic Liquid Multi-Step Extraction (IL)

The utilization of an ionic liquid (1-butyl-3-methylimidazolium acetate) represents a specific modality of solid–liquid extraction [53], at a 1:20 ratio, *w*/*v*, to the biomass, which is followed by sequential separative steps (IL1:20). It encompassed an initial weighing of 0.5 g of dry *R. okamurae* into tubes, to which 10 mL of ionic fluid was added. Then, the tubes were placed in a shaking bath at 75 °C for 2 h. The tubes were taken from the bath and centrifuged at 3000× *g* and 20 °C for 10 min. Then, the obtained supernatant was collected into a new tube, to which 25 mL of pure acetone was added. The supernatant + acetone mixture was filtered through Whatman n° 1 filter paper. The obtained filtrate was subjected to evaporation under vacuum using a Heidolph 401 Digital rotary evaporator (Heidolph Instruments GmbH & Co. KG, Schwabach, Germany), operated at a temperature of 40 °C and a pressure of 400 mbar. After this step, the concentrated filtrate was precipitated using 25 mL of Milli-Q water. The tubes were then placed in the refrigerator at 4 °C overnight under a nitrogen atmosphere. The following day, the mixture was filtered through a Whatman n° 1 filter paper. This last filtrate was transferred to new tubes and stored in the refrigerator at 4 °C until analysis.

#### 4.3.5. Ultrasound-Assisted Extraction (UA)

Dry *R. okamurae* biomass was also used in this solid–liquid extraction technique, coupling two solvents (water and ethanol:water, 70%, *v*/*v*) added either at a 1:10 or 1:20 ratio, *w*/*v*, to the biomass (UAw1:10, UAw1:20, UAew1:10, and UAew1:20), and ultrasound treatment [54]. In this extraction procedure, 5 g of dry *R. okamurae* was weighed into a beaker and the required amount of solvent was added to obtain the intended solid–liquid ratio. After extract mixture preparation, all beakers were placed in an Emerson/Bransonic CPX 5800-E ultrasound bath (Emerson Electric Co., Ferguson, St. Louis, MO, USA), applying 2 cycles of 20 min each. This ultrasound bath conveyed a power of 160 W at an ultrasound frequency of 40 kHz. Afterwards, mixtures were transferred to centrifuge tubes and separated into pellet and supernatant at 3000× *g* and 20 °C for 10 min. Finally, the supernatant was transferred to new tubes and stored in the refrigerator at 4 °C until analysis.

### 4.4. Determination of Moisture and Ash and Calculation of Yields

The moisture and ash contents in percentage were determined in accordance with the methods proposed and described by the Association of Official Analytical Chemists (AOAC) [55]. The respective dry matter and mineral matter yields were calculated through the following formulas:

Dry Matter Yield (%) = ((([Dry Matter]ext/100) × Ext. Wt.)/(([Dry Matter]in/100) × In. Wt.)) × 100(1)

where

[Dry Matter]ext—Dry matter content in % in the attained extract;

[Dry Matter]in—Dry matter content in % in the initial *R. okamurae* biomass;

Ext. Wt.—Weight of attained extract in g;

In. Wt.—Weight of the initial *R. okamurae* biomass used in the extraction in g.

Mineral Matter Yield (%) = ((([Ash]ext/100) × Ext. Wt.)/(([Ash]in/100) × In. Wt.)) × 100(2)

where

[Ash]ext—Mineral matter (ash) content in % in the attained extract;

[Ash]in—Mineral matter (ash) content in % in the initial *R. okamurae* biomass;

Ext. Wt.—Weight of attained extract in g;

In. Wt.—Weight of the initial *R. okamurae* biomass used in the extraction in g.

All final results were expressed on the basis of dry matter terms for the purpose of comparability regardless of being used for dry or wet route processes.

### 4.5. Total Polyphenol Content

The total polyphenol content was determined by the Singleton and Rossi method using Folin–Ciocalteu reagent [56]. For this purpose, 100 μL of each attained extract (see Section 4.3. Seaweed extraction methodologies) was used in triplicate. Gallic acid (GA) was used as a standard and the phenolic content was expressed as gallic acid equivalents (mg GAE/100 g dw of initial *R. okamurae*) according to a gallic acid calibration curve.

### 4.6. Antioxidant Activity as Measured by DPPH Method

The antioxidant activity was measured by determining the radical scavenging activity using 2,2-diphenyl-1-picrylhydrazyl (DPPH) [57]. A 1 mL volume of the extract (selected for a more thorough characterization of the biological activity) was prepared in triplicate for each sample and 2 mL of DPPH (Sigma, Steinheim, Germany) in 0.15 mM methanolic solution was added and thoroughly mixed. After 30 min of incubation at room temperature in the dark, the absorbance was measured at 517 nm in a Helios Alpha UV/visible light spectrophotometer (Unicam, Leeds, UK). The radical scavenging activity was calculated using the following formula:

% Inhibition = (A0 − Asample)/A0 × 100(3)

where

A0—Absorbance of the blank;

Asample—Absorbance of the sample.

On the basis of the ascorbic acid calibration curve, results were then expressed in mg of ascorbic acid equivalents (AA Eq.) per 100 g dw of seaweed.

### 4.7. Antioxidant Activity as Measured by FRAP Method

The antioxidant activity was also determined by the method of Ferric Reducing Antioxidant Power (FRAP). For this objective, a modified version of Benzie and Strain (1996) [58] was applied. In particular, 100 μL of extract (selected for a more thorough characterization of the biological activity) was used in triplicate. Based on an FeSO_4_ calibration curve, results were calculated as μmol Fe^2+^ equivalent (Eq.)/g dw of seaweed.

### 4.8. Antioxidant Activity as Measured by ABTS Method

The ABTS (2,2′-azino-bis(3-ethylbenzothiazoline-6-sulphonic acid)) radical scavenging activity was determined using the method described by Re et al. (1999) [59]. For this, 20 μL of extract (selected for a more thorough characterization of the biological activity) was used in triplicate. The ABTS radical scavenging activity of the samples was expressed as a percentage of inhibition as follows:

% Inhibition = (A0 − Asample)/A0 × 100(4)

where

A0—Absorbance of the blank;

Asample—Absorbance of the sample.

On the basis of a trolox calibration curve, results were then expressed in μmol of trolox equivalents (Trolox Eq.) per 100 g dw of seaweed.

### 4.9. Anti-Inflammatory Activity

The anti-inflammatory activity of the best—with the highest activity and yield—*R. okamurae* extract was determined. A novel aqueous extract for the anti-inflammatory activity was attained from approximately 200 mg of the extract (previously prepared within the scope of Section 4.3) with 2 mL of Milli-Q water. Afterwards, this extract was subjected to heat treatment (80 °C for 1 h) and then centrifuged (3000× *g* and 4 °C for 10 min). The supernatant was collected and the solvent was evaporated using a vacuum rotary evaporator and a water bath at a temperature of 65 °C. The residue was directly dissolved in 100% dimethyl sulphoxide (DMSO) to prepare a stock solution with a concentration of 10 mg/mL. The extract was tested at 1 mg/mL using a commercial cyclooxygenase (COX) inhibitory screening assay kit, Cayman test kit-560131 (Cayman Chemical Company, Ann Arbor, MI, USA). A 10 μL volume of test extract or DMSO (blank) was used. The results were expressed as the percentage of COX-2 inhibition.

### 4.10. Statistical Analysis

To test the normality and the homogeneity of the data variance, the Kolmogorov–Smirnov test and Cochran’s C-test were used, respectively. The data were analysed by one-way ANOVA distributions using Tukey’s HSD to determine the difference in yields, contents, and biological activities between extraction methods and operational parameters. For all the statistical tests, the significance level (α) was 0.05. The Pearson correlation coefficients were also calculated between the antioxidant activities and the total polyphenol content. All the data analyses were performed using STATISTICA 7 (Stat-sof, Inc., Tulsa, OK, USA, 2003).

## 5. Conclusions

There was a substantial degree of overlap between yields and total polyphenol contents in the extracts, thus facilitating the process of identification of the best extractive procedures to apply to *R. okamurae* for further characterization. With respect to the dry matter yield, values differed widely, especially as a function of the type of extractive methodology. Using water or 70% ethanol in mechanical homogenization improved yields (24.4–29.8%). There was a better extractive performance in the Hew trials, especially with a 1:20, *w*/*v*, biomass–solvent proportion, than in the Hw ones. In the case of overnight agitation, 70% ethanol produced results (24.5–28.3%) similar to those found in the homogenized extracts. However, in the former, the best biomass–solvent proportion was 1:10, *w*/*v*. The mineral matter yield presented some specific patterns that partially differed from the dry matter yield. In any case, homogenization using water and 70% ethanol led to yields between 59.6 ± 1.1% (Hew1:20) and 82.3 ± 0.1% (Hw1:20). Water extracts had higher mineral matter yields than the 70% ethanol extracts. The highest total polyphenol level was attained in the Hew1:20 trial, 310.7 ± 22.1 mg GAE/100 g dw. High levels were also attained by overnight agitation (240.0–260.7 mg GAE/100 g dw) or ultrasound-assisted extraction (192.1–236.5 mg GAE/100 g dw).

After assessing these results, the overnight agitation (OAew1:10) and homogenization (Hew1:20) batches were selected for further characterization as the best extracts. Considering DPPH, homogenization (Hew1:20) led to higher antioxidant activity than overnight agitation (OAew1:10), 40.8 ± 0.5 mg AA Eq./100 g dw vs. 18.8 ± 0.1 mg AA Eq./100 g dw. The Hew1:20 extract also had higher FRAP and ABTS values than the other one. On the other hand, the OAew1:10 extract had relevant anti-inflammatory activity (48.3 ± 7.1% of COX-2 inhibition).

Therefore, it was possible to compare different technologies and parameters for a maximization of yields and biological activity in *R. okamurae* extracts through this study. In addition, results showed the potential of *R. okamurae* for pharmacological purposes, especially in the anti-inflammatory area, due to the presence of bioactive compounds. Given the negative effects of *R. okamurae* on the European Coast, this may be a way to support commercially the wild harvest of this species, thus helping the environment by reducing current ecosystem pressures on the coastal economy. The performed experimental work enabled us to rank technologies and conditions for a rapid upscaling of future industrial processing of this seaweed as raw material.

## Figures and Tables

**Table 1:** Total dry matter and mineral matter yields (in %) based on the respective initial contents in the biomass of *Rugulopteryx okamurae* subjected to different extraction methods, including variable operational parameters for each method.

Extraction Method	Operational Parameter	Code	Dry Matter Yield (%)	Mineral Matter Yield (%)
Overnight Agitation (OA)	Biomass–Water 1:10 (*w*/*v*)	OAw1:10	20.7 ± 0.1 d	64.7 ± 0.4 d
	Biomass–Water 1:20 (*w*/*v*)	OAw1:20	21.1 ± 0.1 de	56.2 ± 3.3 c
	Biomass–Ethanol 70% 1:10 (*w*/*v*)	OAew1:10	28.3 ± 0.0 fg	54.0± 4.2 c
	Biomass–Ethanol 70% 1:20 (*w*/*v*)	OAew1:20	24.5 ± 0.0 e	36.4 ± 0.3 b
Mechanical Homogenization (H)	Biomass–Water 1:10 (*w*/*v*)	Hw1:10	24.4 ± 0.2 e	71.0 ± 0.4 e
	Biomass–Water 1:20 (*w*/*v*)	Hw1:20	26.3 ± 0.1 ef	82.3 ± 0.1 f
	Biomass–Ethanol 70% 1:10 (*w*/*v*)	Hew1:10	27.8 ± 0.0 f	62.5 ± 1.3 cd
	Biomass–Ethanol 70% 1:20 (*w*/*v*)	Hew1:20	29.8 ± 0.2 g	59.6 ± 1.1 cd
	Biomass–Ethyl Acetate 1:10 (*w*/*v*)	Hea1:10	5.7 ± 0.0 b	0.0 ± 0.0 a
	Biomass–Ethyl Acetate 1:20 (*w*/*v*)	Hea1:20	5.6 ± 0.0 b	0.0 ± 0.0 a
	Biomass–Isoamyl Acetate 1:10 (*w*/*v*)	Hia1:10	5.7 ± 0.0 b	0.0 ± 0.0 a
	Biomass–Isoamyl Acetate 1:20 (*w*/*v*)	Hia1:20	5.3 ± 0.0 b	0.0 ± 0.0 a
pH-Shift (pHS)	Biomass–1 M HCl 1:10 (*w*/*v*)	pHS1:10	14.5 ± 0.2 c	64.8 ± 0.5 d
	Biomass–1 M HCl 1:20 (*w*/*v*)	pHS1:20	13.3± 0.0 c	64.4 ± 3.5 d
Ionic Liquid (IL)	Biomass–Ionic Liquid 1:20 (*w*/*v*)	IL1:20	0.0 ± 0.0 a	0.0 ± 0.0 a
Ultrasound Agitation (UA)	Biomass–Water 1:10 (*w*/*v*)	UAw1:10	19.7 ± 0.0 cd	60.3 ± 5.3 cd
	Biomass–Water 1:20 (*w*/*v*)	UAw1:20	20.9 ± 0.3 de	53.4 ± 2.0 c
	Biomass–Ethanol 70% 1:10 (*w*/*v*)	UAew1:10	19.6 ± 0.0 cd	33.9 ± 1.1 b
	Biomass–Ethanol 70% 1:20 (*w*/*v*)	UAew1:20	21.6 ± 0.0 de	26.9 ± 2.4 b

Values are presented as the average ± standard deviation. Different lowercase letters within a column correspond to significant differences (*p* < 0.05) between the different extraction methodologies and conditions.

**Table 2:** Total polyphenol contents (in mg GAE/100 g dry weight of seaweed) in the various extracts attained from the biomass of *Rugulopteryx okamurae* subjected to different extraction methods, including variable operational parameters for each method.

Extraction Method	Operational Parameter	Code	Total Polyphenol Content (mg GAE/100 g dw of Seaweed)
Overnight Agitation (OA)	Biomass–Water 1:10 (*w*/*v*)	OAw1:10	118.5 ± 19.1 d
	Biomass–Water 1:20 (*w*/*v*)	OAw1:20	170.8 ± 0.2 f
	Biomass–Ethanol 70% 1:10 (*w*/*v*)	OAew1:10	260.7 ± 7.5 h
	Biomass–Ethanol 70% 1:20 (*w*/*v*)	OAew1:20	240.0 ± 12.6 g
Mechanical Homogenization (H)	Biomass–Water 1:10 (*w*/*v*)	Hw1:10	127.2 ± 1.5 de
	Biomass–Water 1:20 (*w*/*v*)	Hw1:20	157.0 ± 8.6 ef
	Biomass–Ethanol 70% 1:10 (*w*/*v*)	Hew1:10	255.6 ± 12.8 gh
	Biomass–Ethanol 70% 1:20 (*w*/*v*)	Hew1:20	310.7 ± 22.1 i
	Biomass–Ethyl Acetate 1:10 (*w*/*v*)	Hea1:10	83.4 ± 9.4 bc
	Biomass–Ethyl Acetate 1:20 (*w*/*v*)	Hea1:20	148.3 ± 22.0 e
	Biomass–Isoamyl Acetate 1:10 (*w*/*v*)	Hia1:10	114.1 ± 19.5 cd
	Biomass–Isoamyl Acetate 1:20 (*w*/*v*)	Hia1:20	167.8 ± 7.3 f
pH-Shift (pHS)	Biomass–1 M HCl 1:10 (*w*/*v*)	pHS1:10	71.6 ± 6.2 b
	Biomass–1 M HCl 1:20 (*w*/*v*)	pHS1:20	96.0 ± 6.4 c
Ionic Liquid (IL)	Biomass–Ionic Liquid 1:20 (*w*/*v*)	IL1:20	31.7 ± 1.7 a
Ultrasound Agitation (UA)	Biomass–Water 1:10 (*w*/*v*)	UAw1:10	116.0 ± 0.5 d
	Biomass–Water 1:20 (*w*/*v*)	UAw1:20	129.0 ± 6.3 de
	Biomass–Ethanol 70% 1:10 (*w*/*v*)	UAew1:10	192.1 ± 8.9 fg
	Biomass–Ethanol 70% 1:20 (*w*/*v*)	UAew1:20	236.5 ± 20.7 g

Values are presented as the average ± standard deviation. Different lowercase letters within a column correspond to significant differences (*p* < 0.05) between the different extraction methodologies and conditions.

**Table 3:** DPPH antioxidant activity (in mg AA Eq./100 g dry weight of seaweed) in selected extracts from *Rugulopteryx okamurae* subjected to optimized extraction methods.

Extraction Method	Operational Parameter	Code	DPPH (mg AA Eq./100 g dw of Seaweed)
Overnight Agitation (OA)	Biomass–Ethanol 70% 1:10 (*w*/*v*)	OAew1:10	18.8 ± 0.1 a
Mechanical Homogenization (H)	Biomass–Ethanol 70% 1:20 (*w*/*v*)	Hew1:20	40.8 ± 0.5 b

Values are presented as the average ± standard deviation. Different lowercase letters within a column correspond to significant differences (*p* < 0.05) between the different extraction methodologies.

**Table 4:** FRAP antioxidant activity (in μmol Fe^2+^ Eq./g dry weight of seaweed) in selected extracts from *Rugulopteryx okamurae* subjected to optimized extraction methods.

Extraction Method	Operational Parameter	Code	FRAP (μmol Fe^2+^ Eq./g dw of Seaweed)
Overnight Agitation (OA)	Biomass–Ethanol 70% 1:10 (*w*/*v*)	OAew1:10	9.4 ± 0.4 a
Mechanical Homogenization (H)	Biomass–Ethanol 70% 1:20 (*w*/*v*)	Hew1:20	15.7 ± 0.7 b

Values are presented as the average ± standard deviation. Different lowercase letters within a column correspond to significant differences (*p* < 0.05) between the different extraction methodologies.

**Table 5:** ABTS antioxidant activity (in μmol Trolox Eq./100 g dry weight of seaweed) in selected extracts from *Rugulopteryx okamurae* subjected to optimized extraction methods.

Extraction Method	Operational Parameter	Code	ABTS (μmol Trolox Eq./100 g dw of Seaweed)
Overnight Agitation (OA)	Biomass–Ethanol 70% 1:10 (*w*/*v*)	OAew1:10	1034.5 ± 71.7 a
Mechanical Homogenization (H)	Biomass–Ethanol 70% 1:20 (*w*/*v*)	Hew1:20	1489.2 ± 107.6 b

Values are presented as the average ± standard deviation. Different lowercase letters within a column correspond to significant differences (*p* < 0.05) between the different extraction methodologies.

**Table 6:** Matrix of Pearson correlation coefficients between antioxidant activities and polyphenol content for the extracts from *Rugulopteryx okamurae*.

Activity or Phenolic Content	Phenolic Content	DPPH	FRAP	ABTS
Phenolic content	1.00	0.87	0.93	0.91
DPPH	0.87	1.00	0.99	0.95
FRAP	0.93	0.99	1.00	0.97
ABTS	0.91	0.95	0.97	1.00

## Data Availability

The raw data supporting the conclusions of this article will be made available by the authors on request.

## Abbreviations

The following abbreviations are used in this manuscript:

AA Eq.	Ascorbic Acid Equivalent
ABTS	2,2′-azino-bis(3-ethylbenzothiazoline-6-sulphonic acid)
COX	Cyclooxygenase
DPPH	2,2-diphenyl-1-picrylhydrazyl
dw	Dry Weight
ea	Ethyl Acetate
ew	Ethanol–Water 7:3
FRAP	Ferric Reducing Antioxidant Power
GAE	Gallic Acid Equivalent
H	Homogenization
HSD	Honestly Significant Difference
ia	Isoamyl Acetate
IL	Ionic Liquid Multi-Step Extraction
μmol Fe2+ Eq.	Micromole Iron (II) Equivalent
OA	Overnight Agitation
pHS	pH-Shift Extraction
Trolox Eq.	Trolox Equivalent
UA	Ultrasound-Assisted Extraction
w	Water
w/v	Weight/Volume
